# Novel Pharmacological Therapies for the Management of Hyperlipoproteinemia(a)

**DOI:** 10.3390/ijms241713622

**Published:** 2023-09-03

**Authors:** Constantine E. Kosmas, Maria D. Bousvarou, Evangelia J. Papakonstantinou, Donatos Tsamoulis, Andreas Koulopoulos, Rogers Echavarria Uceta, Eliscer Guzman, Loukianos S. Rallidis

**Affiliations:** 1 Division of Cardiology, Department of Medicine, Montefiore Medical Center, Bronx, NY 10467, USA; eliscer@hotmail.com; 2 Cardiology Clinic, Cardiology Unlimited, PC, New York, NY 10033, USA; rogers_eu@hotmail.com; 3 School of Medicine, University of Crete, 710 03 Heraklion, Greece; marabous60@gmail.com (M.D.B.); andrewkoul98@gmail.com (A.K.); 4 General Directorate of Public Health and Social Welfare, Attica Region, 115 21 Athens, Greece; lpapakon777@gmail.com; 5 First Department of Internal Medicine, Thriasio General Hospital of Eleusis, 196 00 Athens, Greece; donatostsamoulis@gmail.com; 6 2nd Department of Cardiology, Medical School, National and Kapodistrian University of Athens, University General Hospital ATTIKON, 124 62 Athens, Greece; lrallidis@gmail.com

**Keywords:** lipoprotein(a), hyperlipoproteinemia(a), cardiovascular disease, antisense oligonucleotides, pelacarsen, small interfering RNAs, olpasiran

## Abstract

Lipoprotein(a) [Lp(a)] is a well-established risk factor for cardiovascular disease, predisposing to major cardiovascular events, including coronary heart disease, stroke, aortic valve calcification and abdominal aortic aneurysm. Lp(a) is differentiated from other lipoprotein molecules through apolipoprotein(a), which possesses atherogenic and antithrombolytic properties attributed to its structure. Lp(a) levels are mostly genetically predetermined and influenced by the size of LPA gene variants, with smaller isoforms resulting in a greater synthesis rate of apo(a) and, ultimately, elevated Lp(a) levels. As a result, serum Lp(a) levels may highly vary from extremely low to extremely high. Hyperlipoproteinemia(a) is defined as Lp(a) levels > 30 mg/dL in the US and >50 mg/dL in Europe. Because of its association with CVD, Lp(a) levels should be measured at least once a lifetime in adults. The ultimate goal is to identify individuals with increased risk of CVD and intervene accordingly. Traditional pharmacological interventions like niacin, statins, ezetimibe, aspirin, PCSK-9 inhibitors, mipomersen, estrogens and CETP inhibitors have not yet yielded satisfactory results. The mean Lp(a) reduction, if any, is barely 50% for all agents, with statins increasing Lp(a) levels, whereas a reduction of 80–90% appears to be required to achieve a significant decrease in major cardiovascular events. Novel RNA-interfering agents that specifically target hepatocytes are aimed in this direction. Pelacarsen is an antisense oligonucleotide, while olpasiran, LY3819469 and SLN360 are small interfering RNAs, all conjugated with a N-acetylgalactosamine molecule. Their ultimate objective is to genetically silence LPA, reduce apo(a) production and lower serum Lp(a) levels. Evidence thus so far demonstrates that monthly subcutaneous administration of a single dose yields optimal results with persisting substantial reductions in Lp(a) levels, potentially enhancing CVD risk reduction. The Lp(a) reduction achieved with novel RNA agents may exceed 95%. The results of ongoing and future clinical trials are eagerly anticipated, and it is hoped that guidelines for the tailored management of Lp(a) levels with these novel agents may not be far off.

## 1. Introduction

Lipid metabolism requires a homeostatic balance between synthesis, circulation, storage and degradation of lipids [1]. Derangement in the metabolism of cholesterol and triglycerides (TG), which are the dominant lipids of clinical significance, provoke a clinical condition known as dyslipidemia. As its etymology suggests, dyslipidemia is defined as abnormal serum lipid levels, constituting one of the most common chronic conditions that modern healthcare professionals face with regard to proper management [2]. The World Health Organization (WHO) estimates that dyslipidemia was responsible for 2.6 million deaths (4.5% of total) and 29.7 million disability-adjusted life years (DALYs) (2.0% of total) in 2016 on a global scale [3]. Its prevalence is estimated to be 39% worldwide and higher in developed countries [4]. The 2017 Global Burden of Disease Study (GBD) pointed out that increased plasma cholesterol levels tend to correlate with socioeconomic development [5]. In the US, the prevalence of dyslipidemia among adults over 20 years old was found to be 45.4% in 2015–2016 [6].

Dyslipidemia is defined as elevated serum concentrations of total cholesterol (TC), low-density lipoprotein cholesterol (LDL-C) and/or TG; a low concentration of high-density lipoprotein cholesterol (HDL-C); or a combination of these elements, although there are more lipoproteins involved, namely very-low-density lipoprotein cholesterol (VLDL-C), intermediate-density lipoprotein cholesterol (IDL-C) and lipoprotein(a) (Lp(a)). LDL-C is considered the main atherogenic component, but non-HDL-C is a more precise index of atherogenicity and comprises the entirety of atherogenic lipoproteins, namely VLDL-C; IDL-C remnants; and Lp(a), the protagonist of the present review article [2]. Epidemiologic studies have established the causative link between atherosclerotic cardiovascular disease (ASCVD) risk and elevated LDL-C levels and low HDL-C levels. In addition, numerous epidemiologic studies (for instance, the ARIC study [7]), Mendelian randomization studies and randomized controlled trials (RCTs) have been taken fully into account in the development of firm, evidence-based guidelines [8,9,10]. Moreover, it is noteworthy that the literature presents dyslipidemia as one of the pathophysiological factors linking insulin resistance (IR) with cardiovascular disease (CVD) [11]. Remarkably, CVD is the principal cause of death worldwide, with a staggering 17.9 million losses in 2019 alone and an estimated loss of 22.2 million lives by 2030 [6].

The prevailing guidelines for classification of CVD risk incorporate the primary established risk factors associated with CVD, including age, plasma apo-B-containing lipoproteins (primarily LDL-C), hypertension, cigarette smoking and diabetes mellitus. These guidelines also consider risk predictors, factors that can modify risk, clinical conditions that might elevate the susceptibility to CVD and, most importantly, the presence or absence of established CVD. Utilizing these criteria, individuals are categorized as having low, moderate, high or very high risk, which subsequently guides the appropriate level of medical intervention [8,9]. In the present review article, Lp(a) is the main subject of interest.

Lipoprotein(a) is strongly associated with CVD [12,13,14,15]. A plethora of studies demonstrate that Lp(a) is closely linked to coronary heart disease (CHD) [16], peripheral artery disease (PAD) [17], stroke [18], cardiac valve calcification [19,20,21,22,23] and abdominal aortic aneurysm [24]. Lp(a) is synthesized in hepatocytes, and its structure closely resembles that of LDL-C. It consists of apolipoprotein B100 (ApoB-100) encircling a lipid core of cholesterol esters (CEs) and an apolipoprotein(a) [Apo(a)], the latter being covalently connected to the ApoB-100 molecule via a single disulfide bond. Apo(a) is the differentiator between Lp(a) and the other lipoproteins, aids in Lp(a) measurement and shares homologous structures with plasminogen (named kringles (K). Plasminogen has five different kringle domains (KI-KV), whereas the LPA gene, which encodes the Apo(a) protein, is characterized by domains KIV and KV, including 10 types of KIV domains: one to forty copies of KIV_2_ and single copies of KIV_1_ and KIV_3–10_. Proatherogenic and proinflammatory oxidized phospholipids circulating in plasma bind on KIV_10_, along with their addition in the lipid phase of the particle, which may partly be responsible. Other kringle types promote certain interactions with foam cells that result in local inflammatory response and further formation of atherosclerotic plaque [12,25,26]. The structural similarity of Lp(a) with plasminogen and tissue plasminogen activator (t-PA) promotes the inhibition of fibrinolysis, influencing the risk for development of ASCVD [27]. Plasma levels of Lp(a) are predominantly genetically predetermined (>90%) rather than affected by lifestyle, and the size of the apo(a) isoform, resulting from the number of KIV_2_ copies, is crucially involved. As a general rule, smaller LPA gene variants encode smaller apo(a) isoforms in greater quantity, as opposed to larger gene variants. The easier secretion of smaller isoforms may directly elevate the Lp(a) plasma concentration, leading to increased CVD risk [25]; in fact, Mendelian randomization studies provide evidence that smaller isoforms and high Lp(a) plasma levels constitute independent causal risk factors for CHD [28]. Overall, Lp(a) is associated with practically every step in the pathogenesis of atherosclerosis and has thrombogenic properties. On the other hand, evidence demonstrates hepatic and renal involvement in the catabolism of Lp(a) [25].

The LPA gene polymorphisms generate highly variable levels of circulating Lp(a) among the population, ranging from <1 mg/dL to >1000 mg/dL [29]. In a recent large study analyzing over 530,000 patients across the US, 35% had Lp(a) >30 mg/dL, and 24% had Lp(a) > 50 mg/dL. These two values represent the upper thresholds for normal Lp(a) levels in the US and Europe, respectively [30]. The incidence of Lp(a) levels > 30 mg/dL is estimated to be between 7% and 26% in the European population [31]. The European Atherosclerosis Society (EAS) 2022 Consensus Statement for Lipoprotein(a) recommends measuring Lp(a) at least once a lifetime in adults in order to identify those with elevated cardiovascular risk. Screening is suggested for young people with high Lp(a) and no other risk factors, those with a history of ischemic stroke and those with a family history of premature ASCVD. Continuous testing is recommended for cases of familial hypercholesterolemia and for individuals with a family history of very high Lp(a) levels. In general, situations such as a family history of premature ASCVD in the absence of prominent, typical risk factors, as well as instances of recurrent CVD events despite optimal management, are indicative of the need for Lp(a) measurement. This measurement can potentially enhance the prediction and improve the classification of CVD risk. Elevated plasma Lp(a) levels further suggest a potential requirement for more aggressive management of the modifiable CVD risk factors. Nevertheless, Lp(a) measurement requires better standardization and harmonization [32,33].

Considering the fact that elevated levels of lipoprotein(a) or hyperlipoproteinemia(a) represent an independent causal risk factor for CVD strongly influenced by genetics, it is crucial to explore pharmacological approaches for to reduce Lp(a). Several agents have been previously suggested and utilized to address elevated Lp(a) but with limited clinical efficacy. In this narrative review article, we provide an overview of those agents and primarily concentrate on novel pharmacological agents currently undergoing preclinical and clinical trials that specifically target Lp(a).

## 2. Lipoprotein(a): How Did We Attempt to Manage Its High Levels Hitherto?

### 2.1. Niacin

Niacin (or vitamin B3) has been acknowledged since the 1950s for its potential antidyslipidemic properties. A recommended dosage range of 1–3 gr of nicotinic acid per day has been used for the management of dyslipidemia when combined with statins [34]. Concerning Lp(a), niacin’s suggested mechanism of action includes downregulation of LPA promoter activity and a reduction in the synthesis of apoB-100 in Lp(a) [35]. A meta-analysis of 14 RCTs involving 9000 patients reported that extended-release niacin (ERN) curtailed lipoprotein(a) levels by 23%, although not in a dose-dependent manner [36]. Furthermore, when combined with statins, niacin was found to substantially reduce carotid intima-media thickness (CIMT) compared to high-intensity statins alone, statins with ezetimibe, and moderate/low-intensity statins, with a mean relative rank of 1.7 [37].

Data emerging in the last decade have raised concerns about the efficacy of niacin as an effective pharmacological agent in reducing CVD risk. A recent meta-analysis and systematic review of 119 clinical trials suggests that although ERN is approved by the Food and Drug Administration (FDA) as a monotherapy for dyslipidemia, its indications are based on findings from older trials and may not align with contemporary and more efficient patient management strategies. Additionally, the occurrence of adverse events limits its widespread use [38]. Moderate-to-high-quality evidence from a Cochrane review that included 23 RCTs does not suggest a significant improvement in cardiovascular events or mortality with niacin, indicating that niacin should not be considered a preventive pharmacological option for CVD [39].

The Atherothrombosis Intervention in Metabolic Syndrome with Low HDL/High Triglycerides: Impact on Global Health Outcomes (AIM-HIGH) trial, which investigated the efficacy of niacin alone or with simvastatin compared to placebo, was discontinued due to a lack of evidence demonstrating efficacy [40]. A substudy based on this trial concluded that niacin does not modify CVD risk associated with elevated Lp(a) levels [41]. Another extensive randomized, placebo-controlled trial, the Heart Protection Study 2-Treatment of HDL to Reduce the Incidence of Vascular Events (HPS2-THRIVE) trial, assessed the efficacy of niacin/laropiprant in over 25,500 participants at high risk for CVD events who were already on simvastatin with or without ezetimibe. The results of this trial did not show any benefit of adding niacin to the standardized cholesterol-lowering regimen [42]. Niacin administration also induced dermatological, gastrointestinal and musculoskeletal adverse events, with a significant increase in the risk of myopathy and alanine transferase (ALT) levels, ultimately leading to the discontinuation of niacin therapy [43,44].

The effectiveness of ERN appears to be influenced by various LPA alleles, although the evidence is controversial. Another substudy of the HPS2-THRIVE trial concluded that niacin led to an average reduction of 30% in Lp(a) levels, but the results varied significantly based on plasma levels of Lp(a) and the size of the apo(a) isoform. The largest isoform sizes (corresponding to lower Lp(a) levels) showed a reduction of 50% in percentage and 4 nmol/L in absolute terms, whereas the smallest isoform sizes (corresponding to higher Lp(a) levels) demonstrated a reduction of 16% and 30 nmol/L, respectively [45]. On the contrary, the AIM-HIGH trial showed a greater reduction in Lp(a) levels among individuals with higher baseline Lp(a) levels [38], and another separate clinical trial demonstrated an average Lp(a) reduction of 28% in male subjects with low-molecular-weight (LMW) apo(a) isoforms, with no significant effects observed in high-molecular-weight isoforms [46].

A recent case report involving individuals with Lp(a) levels exceeding 300 nmol/L demonstrated a 63% reduction in Lp(a) with niacin treatment compared to a 3.9% reduction with aggressive statin therapy. Genetic tests were conducted, revealing a specific variant near the LPA promoter gene that seemed to enhance the effectiveness of niacin treatment, highlighting the importance of precision medicine in niacin therapy [47]. More recently, the use of novel niacin receptor agonists has been proposed as a promising strategy for the treatment of dyslipidemia, including hyperlipoproteinemia(a) [48].

### 2.2. Statins

Statins constitute a fundamental therapeutic pharmacological class of agents for the management of dyslipidemia. They inhibit 3-hydroxy-3-methylglutaryl-coenzyme A (HMG-CoA) reductase and possess additional anti-inflammatory and immunotropic activities [49]. Over the past four decades, research has validated their efficacy in the reduction of LDL-C, which is an independent risk factor for atherogenesis and ASCVD. Their safety profile allows for their administration in numerous candidates for both primary and secondary prevention [50]. Statin treatment effectively reduces total TC and TG levels and increases HDL-C levels [51]. Furthermore, statin therapy safely decreases the 5-year incidence of major cardiovascular events [52]. As a matter of fact, the US Preventive Services Task Force (USPSTF) recently recommended the use of statins for individuals aged 40–75 years with 10% or greater 10-year risk for CVD and at least one CVD risk factor [53].

Elevated Lp(a) levels have been recognized as an ASCVD risk factor, warranting the initiation or intensification of statin therapy [54]. They remain a causal risk factor even when LDL-C levels are well controlled [55]. However, a study demonstrated that lowering LDL-C levels below 2.5 mmol/L (45 mg/dL) does not significantly reduce the Lp(a)-attributed risk for CVD in a primary prevention setting [56]. A recent riveting study based on 445,744 participants proposed practical clinical guidance that quantitively estimates the appropriate LDL-C reduction to overcome the increased risk for ASCVD associated with hyperlipoproteinemia(a) stratified by age, Lp(a) level and hazard ratio for CVD [57].

A meta-analysis of seven statin trials involving approximately 30,000 patients revealed that elevated lipoprotein(a) levels constitute an independent risk factor for CVD, even for patients on statin therapy [58]. However, evidence from a meta-analysis suggests that statins do not improve Lp(a) plasma levels or reduce the CVD risk related to hyperlipoproteinemia(a) [59]. In fact, there is evidence indicating that statins may actually increase Lp(a) levels by roughly 10–20% [26,60]. Interestingly, there is evidence indicating that this statin-associated increase in Lp(a) levels is observed exclusively in patients with low-molecular-weight (LMW) apo(a) isoforms [61].

Notwithstanding, statins remain essential for the treatment of elevated serum LDL-C levels, which are modifiable. The main rationale for the use of statins in hyperlipoproteinemia(a) is their ability to reduce the LDL-dependent portion of the risk for CVD in patients that have an overall higher CVD risk due to increased Lp(a) levels [26].

### 2.3. Ezetimibe

Ezetimibe is a standard lipid-lowering agent used to manage elevated LDL-C levels in patients with dyslipidemia. It acts by binding to the intestinal transporter, Niemann–Pick C1 Like 1 (NPC1L1) protein, thus inhibiting intestinal cholesterol absorption. This agent is capable of reducing plasma LDL-C levels by up to 20% when administered as a monotherapy. Its effect on Lp(a) has remained undefined until a recent systematic review and meta-analysis of seven RCTs involving 2337 patients was conducted. The results revealed that a daily dosage of 10 mg ezetimibe as a monotherapy for 12 weeks led to a slight reduction in plasma Lp(a) levels by 7.06% compared to a placebo (−7.06% [95% CI −11.95 to −2.18]; *p* = 0.005). However, this reduction, despite being statistically significant, is very minor and does not appear to bear any clinical significance [62].

### 2.4. Aspirin

Aspirin permanently inactivates the cyclo-oxygenase activity of prostaglandin (PGH) synthase, with pleiotropic effects in reducing atherosclerotic complications and colorectal cancer, while it potentially increases the risk of spontaneous bleeding [63,64]. However, the net benefit of aspirin in primary prevention is small and, in some cases (adults 60 years or older or at an increased risk of bleeding), the use of aspirin may even be harmful [65,66,67].

Earlier findings regarding the efficacy of aspirin indicated that it may lower plasma Lp(a) levels to approximately 80% of the baseline values when the baseline Lp(a) levels are >30 mg/dL. This reduction exceeds the decrease observed in individuals with lower baseline Lp(a) levels and suggests a favorable downregulation of LPA genes with a higher rate of transcription [68].

In the Women’s Health Study (WHS), 25,000 initially healthy participants who were either carriers or non-carriers of a specific LPA allele variant predisposing to hyperlipoproteinemia(a) and increased risk for CVD were randomized to receive low-dose aspirin or placebo. After a 9.9-year follow-up, the results indicated that carriers had double the risk for major adverse cardiovascular events (MACE), although this risk significantly declined with aspirin (RR reduction: 56%; *p* = 0.033). In comparison, non-carriers did not benefit from aspirin (RR reduction: 9%; *p* = 0.30). The observed benefit may stem from the interaction of aspirin with the apo(a) structure of this specific LPA allele variant [69].

Moreover, a turbidimetric assay assessed the WHS, along with two other cohort studies, and concluded that women with serum Lp(a) levels > 50 mg/dL and total cholesterol > 200 mg/dL were at an increased risk for CVD, although Lp(a) provided minimal only improvement in the prediction of cardiovascular risk [70]. In a month-long study involving 25 patients with ischemic stroke, daily administration of 150 mg of aspirin caused a 55.63% decline in serum Lp(a) levels from baseline when baseline Lp(a) levels were >25 mg/dL, as compared to a 26.63% reduction from baseline when baseline Lp(a) levels were <25 mg/dL [71]. In a case report, a 34-year-old male patient with very high serum Lp(a) levels of 212 mg/dL suffered an ischemic stroke attributed to severe carotid artery stenosis and developed post-carotid endarterectomy thrombosis. Aspirin at a dose of 325 mg reduced Lp(a) levels by 15%, whereas atorvastatin showed no improvement [72].

Finally, a recent study analyzed 12,815 genotyped participants ≥ 70 years of age of European ancestry and without prior CVD events enrolled in the ASPREE (ASPirin in Reducing Events in the Elderly) study, who were randomized to receive 100 mg aspirin daily or placebo. After a median follow-up of 4.7 years, the occurrence of MACE was reduced by 1.7 events per 1000 person years in all subjects. However, in the rs3798220-C and high LPA genomic risk score subgroups, aspirin decreased MACE by 11.4 and 3.3 events per 1000 person years, respectively, without an increased risk of clinically significant bleeding [73].

Overall, aspirin can be characterized as moderately beneficial, being a potential therapeutic option that may mostly assist in cases of genotypes that predispose to increased CVD risk.

### 2.5. PCSK9 Inhibitors

Proprotein convertase subtilisin/kexin type 9 (PCSK9) is a protein produced by hepatocytes that binds to specific regions of LDL receptors (LDLR), forming a complex. This complex is then internalized, leading to lysosomal degradation of the LDLR and reducing the clearance of LDL-C from the circulation.

PCSK9 inhibitors such as evolocumab and alirocumab are monoclonal antibodies that inhibit PCSK-9 activity, thus reducing circulating LDL-C levels. These inhibitors represent a potent LDL-C-lowering option. The literature demonstrates that these pharmacological agents can reduce serum LDL-C levels by up to 60% and also decrease lipoprotein(a) levels, thereby reducing the risk of MACE, without any significant safety and tolerability concerns [74,75].

PCSK9 possibly interacts with Lp(a) molecules via apoB-100, and the mechanism of Lp(a) reduction through PCSK9 inhibitors likely involves both Lp(a) synthesis and the clearance rate [76]. In statin-treated patients receiving alirocumab, this reduction may result from upregulation of the hepatic LDLR and/or decreased competition between Lp(a) and LDL particles for the LDLR [77]. Lp(a) primarily binds to mature PCSK9 rather than furin-cleaved PCSK9, and evolocumab increases the mature form of PCSK-9 while halting the increase in plasma Lp(a) after acute myocardial infarction (MI) [78]. In three cohort studies involving a total of 103,083 participants, a specific loss-of-function mutation in PCSK9 was associated with lower Lp(a) and LDL-C levels, as well as with a reduced risk of aortic valve stenosis and MI [79].

In the FOURIER trial, evolocumab reduced Lp(a) levels by a median of 26.9% [80]. In the ODYSSEY OUTCOMES trial, alirocumab decreased Lp(a) by a median of 5 mg/dL, and every 5 mg/dL reduction in Lp(a) was associated with a 2.5% decrease in CVD risk [81]. Another analysis of the same trial suggested that the reduction in Lp(a) resulting from alirocumab may decrease the risk of PAD and potentially lower the risk of venous thromboembolism (VTE) [82]. The benefit was generally more pronounced among patients with higher baseline Lp(a) levels.

Both a clinical trial and a meta-analysis of 10 RCTs from the ODYSSEY program indicated discordance between LDL-C and Lp(a) reduction, suggesting that evolocumab and alirocumab lower Lp(a) through pathways that do not solely involve the LDLR clearance pathway [83,84]. Numerous clinical trials, RCTs and meta-analyses consistently conclude that evolocumab and alirocumab are valuable tools for the management of hypercholesterolemia in general and hyperlipoproteinemia(a) specifically. The absolute reduction in Lp(a) levels is greater when baseline Lp(a) levels are elevated (although the specific baseline levels may vary), and the risk of MACE is reduced, along with Lp(a) reduction induced by PCSK9 inhibitors [85,86,87,88,89,90,91,92].

### 2.6. Inclisiran

Inclisiran is a small interfering RNA (siRNA) that acts by attenuating the hepatic synthesis of PCSK9. A more detailed description of the mechanism of action of siRNAs is provided later on in this review. Briefly, siRNAs utilize nucleotide base pairing complementarity to bind and inhibit the translation of specific messenger RNA (mRNA) gene sequences, thereby silencing gene expression. In the case of inclisiran, the inhibition of PCSK9 production primarily leads to an increase in LDLR on the hepatocellular cell membrane and subsequently enhanced clearance of LDL-C [93].

The reduction in Lp(a) is a collateral beneficial effect of inclisiran, as demonstrated in several ORION trials. The ORION-1 trial reported reductions of 15% to 26% in Lp(a) levels. In the ORION-9, -10 and -11 trials, inclisiran decreased Lp(a) levels by 13.5%, 21.9% and 18.6% from baseline to day 540, respectively. Notably, a prespecified analysis of the ORION-11 trial demonstrated a significant reduction in Lp(a) levels by 28.5% at day 540. The placebo groups in these trials showed negligible reductions or even a slight increase in Lp(a) levels [94].

Inclisiran was initially approved in December 2020 by the European Medicines Agent (EMA) for use in the EU, intended for adult patients with primary hypercholesterolemia (both heterozygous familial and non-familial) or mixed dyslipidemia [95]. Later on, in December 2021, the FDA approved inclisiran for adults with ASCVD or heterozygous FH who require further reduction in LDL-C [96]. More recently, in July 2023, the FDA granted an extended indication for inclisiran, allowing its use for patients at increased risk of CVD and presenting with elevated LDL-C levels and comorbidities, such as hypertension and DM, even without a prior cardiovascular event [97]. In all cases, inclisiran is intended to complement proper dietary modifications and statin therapy [95,96,97,98]. The recommended initial dosage is 284 mg administered subcutaneously repeated after 3 months, followed by subsequent biannual administrations (every 6 months) for maintenance. This dosing schedule promotes compliance and leads to improved treatment adherence [93,98,99]. An analysis of patient-level data from the ORION-9, -10 and -11 trials suggests that the inclisiran regimen described above results in a reduction in the composite end point of MACE over an 18-month period [100].

Two ongoing clinical trials are awaited to evaluate the clinical outcomes of inclisiran regarding MACE reduction. The first one is ORION-4 (ClinicalTrials.gov Identifier: NCT03705234), which is a phase 3, double-blind, randomized, placebo-controlled study enrolling 15,000 participants aged 55 years or older with pre-existing ASCVD. The intervention includes the administration of 300 mg inclisiran sodium or placebo on the day of randomization, at 3 months, then every 6 months. The estimated primary completion date is July 2026, and the estimated study completion date is December 2049 [93,101]. The other ongoing trial is VICTORION-2 PREVENT (ClinicalTrials.gov Identifier: NCT05030428) conducted by Novartis Pharmaceuticals. This is a phase 3, double-blind, randomized, placebo-controlled trial enrolling 16,500 participants aged 40 years or older with established CVD. The intervention includes the administration of 300 mg inclisiran sodium or placebo on the day of randomization, at 3 months and every 6 months. The study started in 2021, with an estimated primary and study completion date of October 2027 [102].

### 2.7. Other Interventions

*Mipomersen* is a well-studied second-generation antisense oligonucleotide (ASO) that inhibits the synthesis of apoB-10- and apoB-100-containing lipoproteins, including lipoprotein(a). It acts on hepatocytes, which produce apoB-100, and is therefore found in high concentrations in the liver [103]. RCTs and meta-analyses have shown that mipomersen is effective in improving the lipid profile, specifically lowering plasma Lp(a) levels, with a median decline of 24.7%. Original studies reported Lp(a) reductions of 21% [104], 22.7% [105], 25.87% [106], 26.4% [107] and 27.7% [108]. Nandakumar et al. observed a 27% enhancement in the fractional catabolic rate of Lp(a) [104], potentially suggesting a favorable impact on CVD risk. However, notable adverse effects, such as injection-site reactions, flu-like symptoms and hepatic steatosis [104,105,106,108], have led to a higher discontinuation rate [97,100]. Mipomersen was approved by the FDA in January 2013 as an orphan pharmacological agent for homozygous familial hypercholesterolemia (HoFH), but it carries a black-box warning for hepatotoxicity [109]. In contrast, the European Medicines Agency (EMA) rejected the drug in 2012 and 2013 due to concerns regarding its side effects [110,111].

*Estrogens* have long been known for their protective effects regarding CVD. Epidemiological findings suggest that premenopausal females are more protected against CVD events as compared to same-age men. E2, or 17-beta-estradiol, is the most common and active form of estrogen, exerting its activity on DNA regulation via estrogen receptors (ER), namely estrogen receptor alpha (ERα), estrogen receptor beta (ERβ) and G protein-coupled receptor GPR30 (G protein-coupled estrogen receptor 1 or GPER). Their complex has been confirmed to have a positive impact on endothelial function; vascular tone; and atheroprotective properties, including, among others, a favorable lipid profile [112].

Women with premature menopause present a greater risk for CVD, and hormone replacement therapy (HRT) does not alleviate CVD risk in older menopausal women unless started within 10 years after menopause [113]. A recent meta-analysis of 17 studies indicated that a shorter reproductive span is associated with a 31% increased risk of stroke and an elevated total risk of CVD [114].

On average, middle-aged women are classified as an intermediate risk category according to the European SCORE charts, with a SCORE ≥ 1% and <5% at 10 years [115]. Oral estrogen administration in low doses significantly decreases LDL-C and Lp(a) levels and increases HDL-C in postmenopausal women. It is more effective than its transdermal form or its combination with progesterone [116,117].

The association of gender-affirming hormone therapy (GAHT) with possible CVD risk modification in transgender women is also intriguing, and relevant results are awaited [118]. HRT and tibolone, a synthetic steroid used for the management of postmenopausal symptoms, seem to lower Lp(a) levels in a heterogeneous manner, ranging from 19.9% to 44% and from 26% to 48%, respectively, with practically no difference between the two options and no differentiation attributed to estrogen dose, type or coadministration with progesterone [119,120].

Additionally, a meta-analysis of 10 clinical trials including 2049 women demonstrated a reduction in Lp(a) levels by an average of 5.92% attributed to antiestrogen therapy [121]. Lastly, a systematic review suggested a disputable decrease in coronary events attributed to HRT-associated Lp(a) reduction [122].

Overall, evidence reported to date indicates that HRT may be useful for the alleviation of postmenopausal symptoms in certain women, who may further benefit in the case of concurrent hyperlipoproteinemia(a). However, HRT is not recommended as a preventive option to reduce risk of CVD [26].

*Cholesteryl ester transfer protein (CETP) inhibitors* (dalcetrapib, anacetrapib, evacetrapib or TA-8995) are pharmacological agents that increase plasma HDL-C levels. Their mechanism of action involves intervening in the interchange of cholesteryl esters and TG between HDL and LDL lipoproteins [123].

Anacetrapib has been shown to reduce Lp(a) by 34.1%, which is attributed to a decline of 41% in the apo(a) production rate [124], while a meta-analysis also confirmed the efficacy of anacetrapib in reducing Lp(a) by a weighted mean difference (WMD) of 13.35% [125]. In a phase 3 RCT, anacetrapib reduced Lp(a) by 43.1% compared to placebo [126]. Anacetrapib has also been shown to increase HDL-C and decrease non-HDL-C, with relative differences of +104% and −18%, respectively [127].

In an ad hoc analysis of the dal-OUTCOMES trial, dalcetrapib was found to modestly but significantly reduce Lp(a) from baseline compared to placebo (−1.7 mg/dL vs. −0.6 mg/dL, *p* < 0.001). [128]. Torcetrapib, another CETP inhibitor, was found to reduce plasma Lp(a) levels by 11.1% [129]. Regarding their effect on cardiovascular outcomes, a meta-analysis of 11 RCTs indicated that CETP inhibitors are not associated with an increase in major adverse cardiovascular events (MACE) (pooled RR: 0.97; 95% CI: 0.91–1.04), with a decreasing, non-statistically significant trend for non-fatal MI (RR: 0.93; 95% CI: 0.87–1.00) and cardiovascular mortality (RR: 0.92; 95% CI: 0.83–1.01) [130]. However, a more recent study that included participants in 2 RCTs of a CETP inhibitor on a background of atorvastatin therapy suggested that evacetrapib and torcetrapib may increase the risk of CHD due to their potential to augment dysfunctional HDL particles. [131].

Thus, given the varying effects of CETP inhibitors on CVD outcomes, ranging from harmful to only marginally favorable [132], CETP inhibitors have not entered into clinical practice.

It is worth highlighting that a recent Mendelian randomization study suggests that achieving a significant reduction of 80% to 90% in Lp(a) levels is necessary to observe a subsequent 15% to 20% decline in CHD. This is in contrast to the reductions of 20% to 35% achieved thus far with the currently available lipid-lowering drugs [133]. It is important to note that the FDA has not approved any pharmacological interventions to date, including those mentioned above, to lower Lp(a) levels, except for lipoprotein apheresis, which has been shown to reduce Lp(a) by 63% post apheresis as compared to pre-apheresis levels [134]. Further analysis of lipoprotein apheresis exceeds the scope of this narrative review.

## 3. Novel RNA Targeting Agents in the Pipeline for the Management of Hyperlipoproteinemia(a)

Since no clinically significant results have been attained with the use of the aforementioned Lp(a)-lowering agents, pharmaceutical companies have resorted to implementing alternative novel technologies in an attempt to reduce lipoprotein(a) to the greatest feasible extent possible, considering its atherosclerosis-related properties. In particular, four novel agents are currently under intensive study: pelacarsen by Novartis/Ionis, olpasiran by Amgen, LY3819469 by Eli Lilly and an emerging agent known as SLN360 by Silence Therapeutics. These agents aim to reduce plasma Lp(a) levels through genetic silencing at a post-transcriptional level [135].

### 3.1. Antisense Oligonucleotides—Pelacarsen

Antisense oligonucleotides (ASOs) are synthetic single-strand oligonucleotides up to 30 bases in length. They target and bind to specific RNAs, hindering their translation through blockage, degradation or, more commonly, cleavage via the ribonuclease H (RNase H) mechanism, following the rule of complementarity. Rapid degradation of ASOs can be circumvented by modifying the phosphate groups, resulting in second-generation ASOs [135,136].

*Pelacarsen or AKCEA-Apo(a)-L_Rx_*, formerly known as ISIS 681257 or IONIS-Apo(a)-L_Rx_, is an intriguing second-generation ASO. It features a trifurcated N-acetylgalactosamine (GalNAc) molecule chemically attached to it. The complex is rapidly taken up by the asialoglycoprotein receptor (ASGPR), which is abundant in hepatocytes, the same cells where Lp(a) production occurs. This uptake ultimately hinders the translation of LPA messenger RNA into apo(a) [135,136]. This liver selectivity yields 30 times higher efficacy than pelacarsen’s predecessor molecule without a covalent GalNAc, called IONIS-Apo(a)-R_x_, achieving the same or enhanced results with a 10 times lower dose and improved tolerability [135,137]. Once the ASGPR–pelacarsen complex is taken up into vesicles by the hepatocyte, separation of ASGPR from Gal-NAc occurs. The former is either recycled or degraded, whereas the latter undergoes degradation in lysosomes [138].

Trials have demonstrated the efficacy of ASOs in markedly reducing plasma Lp(a) levels. A phase 2 trial and a phase 1/2a trial compared IONIS-Apo(a)-R_x_ and IONIS-Apo(a)-L_Rx_ (pelacarsen), respectively, with placebo. In the phase 1/2a trial, 58 participants with Lp(a) levels of 75 nmol/L (approximately 35 mg/dL, converted by dividing the value in nmol/L by 2.15; r^2^ = 0.998 for linearity [139]) or higher were randomly assigned to groups receiving multiple ascending doses of pelacarsen (10, 20 or 40 mg) or placebo. On day 36, pelacarsen had effectively reduced plasma Lp(a) levels in a dose-dependent manner, with mean reductions ranging from 66% to 92% (*p* = 0.0007 for all vs. placebo). Overall, pelacarsen demonstrated an excellent safety profile with, no injection-site adverse events reported, in comparison to IONIS-Apo(a)-Rx (12% of participants experienced injection-site reactions) [137].

In a phase 2 double-blind dose-ranging RCT, 286 patients with established CVD and elevated Lp(a) levels of at least 60 mg/dL were recruited and assigned to receive 20, 40 or 60 mg of pelacarsen every 4 weeks; 20 mg ever 2 weeks; 20 mg every week; or placebo subcutaneously for 6 to 12 months. Pelacarsen caused a dose-dependent reduction in plasma Lp(a) levels, with mean decreases ranging from 35% to 80% depending on the dose, compared to a 6% reduction in the placebo group (*p* values ranged from 0.003 to <0.001). Injection-site reactions were noted in 27% of the pelacarsen recipients, with erythema being the most common reaction. No significant differences were observed between the pelacarsen and placebo groups regarding adverse events (90% versus 87%, respectively), although 10% of the pelacarsen group reported serious adverse events. Adverse events included myalgia, influenza-like symptoms and urinary tract infections. There were no significant differences between pelacarsen and placebo regarding platelet count, hepatic and renal function or influenza-like symptoms. Adverse events were not dose-dependent [140].

A study based on the previous trial provided further valuable findings. Researchers described a novel method for the direct measurement of Lp(a) cholesterol [Lp(a)-C] rather than using the Dahlén formula (LDL-C_corr_), as LDL-C measurements, in general, include LDL-C and Lp(a)-C. At the primary analysis time point, pelacarsen demonstrated a dose-dependent reduction in Lp(a)-C (29% to 67%) as compared to a 2% reduction in the placebo group. The statistically significant changes aligned with the previously measured Lp(a) molar concentration. LDL-C_corr_ appeared to be nearly 13–16 mg/dL lower than routinely measured LDL-C in hyperlipoproteinemia(a), with the rest being undetected Lp(a)-C, raising concerns regarding the main etiological factor of MACE in such cases. As mentioned above, standard LDL-C measurement includes Lp(a)-C, suggesting an association between lower levels of LDL-C and Lp(a) reduction. The authors proposed this alternative laboratory approach to improve clinical management and decision making. Other Lp(a) components, such as oxidized phospholipids covalently bound with apoB, were also reduced [141].

In a randomized, double-blind, placebo-controlled trial, 29 healthy Japanese participants were recruited and treated with single doses (20, 40 or 80 mg) and multiple doses of pelacarsen (80 mg every 4 weeks for 4 doses). In the single-dose groups, the mean reductions in Lp(a) compared to placebo were −55.4% (*p* = 0.0008), −58.9% (*p* = 0.0003) and −73.7% (*p* < 0.0001), respectively. In the multiple-dose group, the mean decline varied from −55.8% to −106.2% on different days (mean reduction compared to placebo on days 29, 85, 113, 176 and 204 was −84.0% (*p* = 0.0003), −106.2% (*p* < 0.0001), −70.0% (*p* < 0.0001), −80.0% (*p* = 0.0104) and −55.8% (*p* = 0.0707), respectively). The peak mean plasma concentrations occurred 4 h after administration, and no significant adverse events were observed [142].

Recently, a phase 1 parallel-group study was completed, focusing on assessing the pharmacokinetics of single-dose pelacarsen in eight subjects with mild hepatic impairment compared to eight healthy participants matched by age, gender and body weight. The results are eagerly awaited [143].

A crucial ongoing phase 3 randomized clinical trial is the Lp(a) HORIZON trial, which started in December 2019 and is estimated to be completed in May 2025. The trial includes 8323 participants who meet specific inclusion criteria, such as having Lp(a) ≥ 70 mg/dL and optimal management of LDL-C with other accompanying CVD factors. The intervention involves monthly subcutaneous administration of 80 mg pelacarsen or placebo for 4 to 5 years. This trial aims to determine whether reducing Lp(a) levels has a clinical benefit in terms of reducing MACE [144].

Another ongoing phase 3 trial recruiting participants in Germany since August 2019 and estimated to be completed in July 2024 aims to evaluate the impact of monthly administration of 80 mg pelacarsen on the reduction in the lipoprotein apheresis rate in 60 participants with hyperlipoproteinemia(a) and established CVD. All participants must have Lp(a) > 60 mg/dL and have been undergoing weekly lipoprotein apheresis for at least one year prior to the intervention [145].

Last but not least, a double-blind, placebo-controlled RCT is scheduled to start in September 2023, with an estimated completion date in 2028. The trial aims to recruit approximately 502 participants aged 50 and above with Lp(a) levels ≥ 125 nmol/L (approximately 58 mg/dL). The purpose of this trial is to assess the efficacy and safety of monthly subcutaneous administration of 80 mg pelacarsen in relation to the calcification process of aortic valves as compared to placebo [146].

Until now, pelacarsen has been the most extensively studied factor for lowering Lp(a) among novel interventions, demonstrating remarkable efficacy in reducing plasma Lp(a) levels with minimal adverse events. Mild injection-site reactions have been the most commonly reported side effects. The eagerly awaited results of pivotal clinical trials will provide valuable insights into the effect of Lp(a) reduction on CVD risk and the potential role of pelacarsen in reducing MACE. These trials aim to establish pelacarsen as a standard clinical strategy for the management of dyslipidemia and, in particular, hyperlipoproteinemia(a). It is noteworthy that pelacarsen effectively lowers Lp(a) levels, regardless of different LPA alleles and isoforms [147].

A summary of the results of the clinical studies pertaining to pelacarsen is shown in Table 1.

### 3.2. Small Interfering RNA (siRNA) Molecules—Olpasiran, LY3819469 and SLN360

Small interfering RNA (siRNA) molecules represent the other side of the coin in post-transcriptional RNA interference. Currently, they are undergoing rigorous investigation in clinical trials, making them promising agents in the future clinician’s arsenal. siRNAs typically consist of duplex RNA molecules that are 21–23 nucleotides in length, comprising the passenger (sense) strand and the guide (antisense) strand. It is crucial for their length to be less than 30 nucleotides; otherwise, siRNAs may be recognized as antigens by innate immune receptors, such as toll-like receptors (TLRs). The duplex RNA interacts with the RNA-induced silencing complex (RISC), leading to the unwinding and subsequent degradation of the passenger strand. The guide RNA is then available to bind to the target mRNA and direct the RISC towards it. The pharmacokinetic and pharmacodynamic properties of siRNAs can be modified through various alterations, including sugar and base modifications, phosphorothioate linkages that replace phosphodiester linages and modifications of the duplex structure [148].

### 3.3. Olpasiran

Olpasiran, which was developed and studied by Amgen, was previously known as AMB-890 or ARO-LPA. It is a synthetic siRNA with a GalNAc attached to it, similar to pelacarsen, and has undergone modifications regarding sugar and phosphate backbone linkages. It is administered subcutaneously [135]. Olpasiran’s mechanism of action involves inhibiting LPA mRNA translation, leading to a subsequent reduction in apo(a) production by hepatocytes and lowering of plasma Lp(a) levels. Preclinical data demonstrate its dose-dependent efficacy in transgenic mice and cynomolgus monkeys, where a single dose achieved a peak reduction of 80% in Lp(a) from baseline levels, which lasted for 5 to 8 weeks [149].

In a phase 1 randomized dose-ascending trial involving 79 patients with elevated Lp(a) levels at enrollment, it was demonstrated that olpasiran at a dose of 9 mg or higher can reduce Lp(a) concentrations by 71% to 97%, on average, lasting for a mean duration of 3 to 6 months [149]. Based on the aforementioned trial, another phase 1 randomized parallel-group study was conducted, involving both healthy Japanese and non-Japanese subjects (27 in total). The study evaluated the effects of olpasiran at single ascending doses in Japanese subjects and at a fixed dose of 75 mg in non-Japanese subjects. The findings of the study assessed the pharmacokinetics of olpasiran and reported a dose-proportional reduction in Lp(a) levels from baseline, ranging from 56% to 99%. These reductions were observed as early as day 4 after administration. Importantly, the effect on Lp(a) did not differ significantly between Japanese and non-Japanese subjects, and no significant adverse events were reported [150].

A phase 2 clinical trial named Olpasiran trials of Cardiovascular Events And lipoproteiN(a) reduction-DOSE finding study [OCEAN(a)-DOSE] randomly assigned 281 patients with a median Lp(a) concentration of 260.3 nmol/L (121 mg/dL) and established atherosclerotic CVD who were on cholesterol-lowering therapy to receive ascending doses of olpasiran every 12 weeks or placebo. The mean reductions, adjusted for placebo, ranged from 70% to 101% (*p* < 0.001 for all comparisons) at the primary end point (week 36) and at the end of the treatment period (week 48). The most commonly reported adverse event was injection-site pain [151,152]. The study provided valuable data that can be extrapolated to ascertain the ideal dosage and the effect of olpasiran on MACE. In fact, a phase 3 trial called the OCEAN(a)-DOSE Outcomes Trial is an expansion of the OCEAN(a)-DOSE study and is currently recruiting. It aims to enroll 6000 participants with Lp(a) concentration ≥ 200 nmol/L and established CVD who will receive olpasiran or placebo. The objective of this trial is to evaluate the effect of olpasiran in reducing MACE, in particular CHD death, myocardial infarction or urgent coronary revascularization [153].

A recently completed phase 1 clinical trial for which published results are not yet available assessed the safety, pharmacokinetics and pharmacodynamics of a single dose of olpasiran in 25 eligible patients with various degrees of hepatic impairment compared to placebo. The primary and secondary outcome measures were evaluated on days 29 and 85 after the administration of olpasiran, respectively [154]. Results of another completed phase 1 open-label, single-dose study on olpasiran assessing it safety profile, pharmacokinetics and pharmacodynamics in 24 Chinese participants with elevated Lp(a) concentration are eagerly awaited [155], and a currently recruiting phase 1 open-label study will evaluate single-dose olpasiran in 32 patients with normal and impaired renal function. The latter study is estimated to be completed in August 2023 [156].

### 3.4. SLN360

SLN360 is another siRNA conjugate with a trifurcated GalNAc molecule. Developed by Silence Therapeutics, this agent targets and inhibits LPA mRNA translation into apo(a) in hepatocytes [135]. SLN360 robustly reduced LPA mRNA molecules in primary human hepatocytes and in healthy cynomolgus monkeys, resulting in a serum Lp(a) reduction of over 95% from baseline for at least 9 weeks after dosing, with the peak effect observed on day 21. The minimally effective dose was found to be 0.3 mg/kg [157]. Additional toxicological analyses in vitro and in vivo demonstrated sufficient evidence regarding safety [158].

In the first ongoing phase 1 study (APOLLO study) involving 32 healthy participants with elevated serum Lp(a) levels (more than 150 nmol/L or approximately 70 mg/dL), the safety, tolerability, pharmacokinetics and pharmacodynamics of SLN360 were assessed. Participants were randomly assigned to receive single ascending doses of SLN360 (30, 100, 300 and 600 mg) or placebo, with the primary outcome being the evaluation of safety, tolerability and the effect on serum Lp(a) levels on day 150. The last follow-up occurred on 29 December 2021. The reduction in Lp(a) levels reached a peak of 98% with the 600 mg dose of SLN360 (30 mg: −46% (95% CI: −64 to −40); 100 mg: −86% (95% CI: −92 to −82); 300 mg: −96% (95% CI: −98 to −89); 600 mg: −98% (95% CI: −98 to −97); placebo: −10% (95% CI: −16 to 1)), and the reduced levels were maintained until day 150 with adequate safety [159].

It is worth mentioning that a phase 2 randomized placebo-controlled trial was recently launched, enrolling 160 participants at high risk for ASCVD with serum Lp(a) levels above 125 nmol/L (58 mg/dL). The objective of this study is to investigate the safety profile, efficacy and tolerability of SLN360. The study began in January 2023 and is estimated to be completed in June 2024 [160].

### 3.5. LY3819469

The latest siRNA-targeting apo(a)-encoding mRNA was developed by Eli Lilly, once again conjugated with GalNAc with proper structural modifications. It is a 2′-o-me, 2′-fluoro and unmodified Dicer siRNA [161]. A phase 1 single-ascending-dose, placebo-controlled study was completed in November 2022. The purpose of the first part of the study was to assess the pharmacokinetics, pharmacodynamics, safety and tolerability profile of LY3819469 in participants with elevated serum Lp(a) levels. The second part of the study focuses on LY3819469 administration in Japanese patients. No results had been published at the time of writing this manuscript [162].

An ongoing phase 2 placebo-controlled trial will evaluate the efficacy and safety of LY3819469 in an estimated 254 participants with elevated serum Lp(a) levels above 175 nmol/L (81 mg/dL) over the course of 20 months. The trial is expected to be finalized in October 2024 [163].

Last but not least, a new phase 1 study is currently recruiting and being designed accordingly in order to evaluate the pharmacokinetics, safety and efficacy of LY3819469 in 28 patients with normal and impaired renal function. The study is estimated to last up to 17 weeks [164].

Similarly to any novel pharmacological agent, limitations are raised concerning the overall clinical efficacy of these novel Lp(a)-targeting agents. While it is acknowledged that a significant reduction in Lp(a) levels must occur for clinical results to be achieved, there is no defined cutoff target value, as is the case with LDL-C [165]. Furthermore, the measurement of Lp(a) levels should be standardized. A clearer understanding of Lp(a) physiology and metabolism is needed, along with clarification of the complex pathophysiological link between Lp(a) and its oxPLs with atherosclerosis and CVD. In addition, Lp(a) levels vary among ethnicities [166]. Novel agents appear to be extremely efficient in reducing Lp(a) levels; however, their long-term clinical efficacy in improving cardiac outcomes will be eventually evaluated by appropriately designed large prospective clinical trials. The results of these trials will enhance our knowledge in the field of Lp(a) and provide the foundation for the establishment of definitive indications for the management of hyperlipoproteinemia(a) in patients of different ages and ethnicities and with specific CVD risk factors or other health conditions.

A summary of the results of the clinical studies pertaining to siRNA molecules is shown in Table 2.

## 4. Conclusions and Future Perspectives

In summary, lipoprotein(a) decisively influences the risk for developing CVD in a proportional manner; the higher the levels, the greater the risk of major atherosclerotic cardiovascular events. While traditional pharmacological approaches have been able to reduce levels to some degree, the clinical significance of this reduction has been limited, and the adverse effects have outweighed the benefits. As a result, the research community has been intensely focused on assessing newer agents for lowering Lp(a) levels. Antisense oligonucleotides and small interference RNAs share the conjugation with a GalNAc molecule and represent promising novel agents that will significantly enhance the armamentarium of modern clinicians. These agents utilize genetic silencing as their primary mechanism of action. At the same time, these newer agents offer patients with elevated serum Lp(a) concentrations the potential for increased protection against the occurrence of major atherosclerotic cardiovascular events compared to contemporary standard lipid management agents. The evidence collected so far supports a satisfactory safety and tolerability profile, along with long-lasting efficacy following subcutaneous single-dose administration of the aforementioned agents.

Pelacarsen, SLN360 and LY3819469 appear promising in providing a clinically meaningful Lp(a) reduction and improving cardiovascular outcomes. However, further large trials assessing cardiovascular outcomes are required and are eagerly anticipated to provide more comprehensive insights and determine whether some of these agents have the potential to become part of everyday clinical practice in the future.

## Figures and Tables

**Table 1:** Clinical trials pertaining to pelacarsen or IONIS-APO(a)L_Rx_.

Trial	Design	Intervention	Results
[137] Viney et al. ClinicalTrial.gov Identifier: NCT02160899	A randomized, multicenter, double-blinded, placebo-controlled phase 2 trial enrolling 64 participants with elevated Lp(a)	Patients were stratified in two cohorts (cohort A: 51 patients with Lp(a) of 125–437 nmol/L; cohort B: 13 patients with Lp(a) ≥ 438 nmol/L) and received subcutaneous ascending doses of IONIS-APO(a)_Rx_ or injections of saline placebo for 12 weeks	On days 85 and 99: In cohort A: IONIS-APO(a)_Rx_ reduced Lp(a) by a mean of 66.8% (SD 20.6, *p* < 0.001 vs. pooled placebo); In cohort B: IONIS-APO(a)_Rx_ reduced Lp(a) by a mean of 71.6% (SD 13.0, *p* < 0.001 vs. pooled placebo); Reported injection-site reactions in 12% of subjects in the IONIS-APO(a)_Rx_ group
[137] Viney et al. ClinicalTrial.gov Identifier: NCT02414594	A randomized, blinded, placebo-controlled phase 1/2a trial enrolling 58 healthy participants with Lp(a) ≥ 75 nmol/L	Participants were random assigned to receive: (i) a single dose of 10–120 mg IONIS-APO(a)L_Rx_ in an ascending-dose design or placebo (3:1 ratio); or: (ii) multiple doses of 10 mg, 20 mg or 40 mg IONIS-APO(a)L_Rx_ in an ascending-dose design or placebo (8:2 ratio)	Single-dose groups: dose-dependent reductions in mean Lp(a) levels in the IONIS-APO(a)-L_Rx_ group on day 30; Multidose groups: mean reductions in Lp(a) of 66% (SD 21.8) in the 10 mg group, 80% (SD 13.7%) in the 20 mg group and 92% (SD 6.5) in the 40 mg group (*p* = 0.0007 for all vs. placebo) on day 36; No injection-site reactions
[140] Tsimikas et al. ClinicalTrial.gov Identifier: NCT03070782	A randomized, double-blind, dose-ranging, placebo-controlled phase 2 study of AKCEA-APO(a)-LRx enrolling 286 patients with Lp(a) levels ≥ 150 nmol/L	AKCEA-APO(a)-LRx administration (20, 40 or 60 mg every 4 weeks; 20 mg every 2 weeks; or 20 mg every week) or saline placebo subcutaneously for 6 to 12 months	At month 6: Mean Lp(a) reductions ranging from 35% to 80% in a dose-dependent manner vs. 6% reduction with placebo (*p* < 0.003 to 0.001); 27% of the intervention group reported injection-site reactions, mostly erythema; No difference between intervention and placebo groups in the incidence of adverse events
[141] Yeang et al.	Study design based on the above phase 2B trial by Tsimikas et al. [140]	Laboratory measurement of Lp(a), application of a pioneer method for the direct measurement of corrected Lp(a) at baseline and week 13, primary analysis at week 25/27 and final analysis at week 69, with LDL-C laboratory measurements further corrected	At the primary analysis time point: dose-dependent reduction in Lp(a) (29% to 67% versus 2% the placebo group); Corrected LDL-C13–16 mg/dL lower than routinely measured LDL-C in hyperlipoproteinemia(a), with the rest being so far undetected Lp(a)–C
[142] Karwatowska-Prokopczuk E et al.	A randomized, double-blind, placebo-controlled trial enrolling 29 healthy Japanese participants	Single ascending doses of pelacarsen (20, 40 or 80 mg) or multiple doses of pelacarsen 80 mg (monthly for four doses)	Single-dose group: mean Lp(a) reductions were −55.4% (*p* = 0.0008), −58.9% (*p* = 0.0003) and −73.7% (*p* < 0.0001) versus placebo; Multiple-dose group: the mean decline varied from −55.8% to −106.2% at different time checkpoints
[143] ClinicalTrials.gov Identifier: NCT05026996	An open-label, single-dose, parallel-group phase 1 study involving 16 patients with mild liver impairment and normal liver function	Single subcutaneous injection of pelacarsen versus placebo	Impact of pelacarsen on mild hepatic impairment Study completed; results are awaited
[144] ClinicalTrials.gov Identifier: NCT04023552	An ongoing randomized, double-blind, placebo-controlled multicenter phase 3 trial enrolling 8323 participants with Lp(a) ≥ 70 mg/dL and optimal management of LDL-C and other accompanying CVD factors	Monthly subcutaneous administration of 80 mg pelacarsen or placebo for 4 to 5 years	End point: impact of pelacarsen-induced Lp(a) reduction on MACE reduction; Estimated completion date: May 2025
[145] ClinicalTrials.gov Identifier: NCT05305664	A randomized, double-blind, placebo-controlled multicenter phase 3 trial currently recruiting 60 participants on weekly lipoprotein apheresis with Lp(a) > 60 mg/dL and CVD risk factors	Monthly subcutaneous administration of 80 mg pelacarsen versus placebo	Evaluation of effect of intervention on lipoprotein apheresis rate; Estimated completion date: July 2024
[146] ClinicalTrials.gov Identifier: NCT05646381	A randomized double-blind, placebo-controlled, multicenter Trial including 502 participants aged ≥ 50 with Lp(a) levels ≥ 125 nmol/L and mild or moderate calcific aortic valve stenosis	Monthly subcutaneous administration of 80 mg pelacarsen versus placebo	Assessment of the efficacy and safety of pelacarsen regarding the aortic calcification process; Study start date: September 2023; Estimated completion date: January 2028
[147] Karwatowska-Prokopczuk E et al.	Evidence from four trials involving pelacarsen in a total of 455 patients	Data analysis involving the prevalence of common LPA isoform alleles and their effect on the efficacy of pelacaesen	Pelacarsen reduces Lp(a) levels independently of different LPA alleles and isoforms

**Table 2:** Clinical trials pertaining to siRNA molecules.

*Olpasiran*			
Trial	Design	Intervention	Results
[149] Koren et al. (clinical part of the study)	A randomized, double-blind, placebo-controlled, single-ascending-dose, phase 1 trial enrolling 79 patients with elevated Lp(a) levels	Single dose of olpasiran versus placebo	Olpasiran at a dose of 9 mg or higher reduced Lp(a) concentrations by 70% to 97%, on average, with a mean duration of 3 to 6 months
[150] Sohn et al.	A randomized, open-label, parallel-design, dose-ascending, phase 1 trial involving 37 healthy Japanese and non-Japanese patients	Japanese: single 3, 9, 75 or 225 mg dose of olpasiran (1:1:1:1 ratio) Non-Japanese: a single 75 mg dose of olpasiran	Dose-proportional reduction in Lp(a) levels from baseline, ranging from 56% to 99%, observed as early as day 4; Effect on Lp(a) did not differ significantly between Japanese and non-Japanese subjects
[151,152] O’ Donohue et al. OCEAN(a)-DOSE study	A randomized, double-blind, placebo-controlled, multicenter dose study in 281 participants with a median Lp(a) concentration of 260.3 nmol/L (121 mg/dL) and established atherosclerotic CVD on cholesterol-lowering therapy	Ascending doses of olpasiran (10 mg every 12 weeks, 75 mg every 12 weeks, 225 mg every 12 weeks or 225 mg every 24 weeks) versus placebo	Mean reductions (adjusted for placebo) ranged from 70% to 101% (*p* < 0.001 for all comparisons) at the primary end point (week 36) and the end of the treatment period (week 48); Most common adverse event: injection-site pain
[153] ClinicalTrials.gov Identifier: NCT05581303 OCEAN(a)-DOSE Outcomes Trial	An ongoing randomized, double-blind, placebo-controlled, multicenter phase 3 study currently recruiting (aim: 6000 participants with Lp(a) concentration ≥ 200 nmol/L and established CVD)	Olpasiran versus placebo	Effect of olpasiran on the risk for coronary heart disease death (CHD death), myocardial infarction or urgent coronary revascularization, matched with placebo; Study start: December 2022; Estimated completion date: December 2026
[154] ClinicalTrials.gov Identifier: NCT05481411	An open-label, single-dose phase 1 trial including 25 patients with mildly, moderately and seriously impaired hepatic function	Olpasiran	Assessment of pharmacokinetics, pharmacodynamics, safety and tolerability of olpasiran on the grounds of hepatic impairment
[155] ClinicalTrials.gov Identifier: NCT04987320	An open-label, single-dose phase 1 clinical trial involving 24 Chinese participants with elevated Lp(a) levels	Single dose of olpasiran	Assessment of pharmacokinetics, pharmacodynamics, safety and tolerability of olpasiran; Results not yet published
[156] ClinicalTrials.gov Identifier: NCT05489614	An ongoing, open-label, single-dose phase 1 study enrolling 32 patients with normal renal function or various degrees of renal impairment	Single dose of olpasiran	Assessment of pharmacokinetics, pharmacodynamics and safety of olpasiran; Estimated completion date: August 2023
***SLN360***			
**Trial**	**Design**	**Intervention**	**Results**
[159] Nissen et al.	An ongoing randomized, double-blind, placebo-controlled, first-in-human phase 1 study including 32 participants with Lp(a) levels ≥ 150 nmol/L who were randomized and received the intervention	Single doses of SLN360 at 30, 100, 300 or 600 mg versus placebo	The reduction in Lp(a) levels reached a peak of 98% with SLN360 at a dose of 600 mg (30 mg: −46% (95% CI: −64 to −40); 100 mg: −86% (95% CI: −92 to −82); 300 mg: −96% (95% CI: −98 to −89); 600 mg: −98% (95% CI: −98 to −97); placebo: −10% (95% CI: −16 to 1)); Reduction maintained until day 150 with satisfactory safety
[160] ClinicalTrials.gov Identifier: NCT05537571	An ongoing randomized, double-blind, placebo-controlled, multicenter phase 2 study enrolling 160 participants with Lp(a) levels above 125 nmol/L at high risk of CVD	SLN360 versus placebo	Evaluation of the safety, efficacy and tolerability of SLN360; Study start: January 2023; Estimated completion date: June 2024
***LY3819469***			
**Trial**	**Design**	**Intervention**	**Results**
[162] ClinicalTrials.gov Identifier: NCT04914546	A two-part, single-ascending-dose, placebo-controlled phase 1 study in 66 healthy participants with elevated Lp(a) concentrations	Single ascending dose of LY3819469 versus placebo	Evaluation of the pharmacokinetics, pharmacodynamics, safety and tolerability of LY3819469 in healthy participants (part A) and in Japanese participants (part B); Results not yet published
[163] ClinicalTrials.gov Identifier: NCT05565742	A randomized, double-blind, placebo-controlled phase 2 study enrolling 254 participants with Lp(a) levels ≥ 175 nmol/L	LY3819469 versus placebo	Evaluation of the efficacy and safety of LY3819469 in the course of 20 months
[164] ClinicalTrials.gov Identifier: NCT05841277	An ongoing, currently recruiting phase 1 study including 28 participants with normal and impaired renal function	LY3819469	Evaluation of pharmacokinetics, efficacy and safety of LY3819469 on the grounds of various degrees of renal function

## Data Availability

All data supporting the findings of this study are available within the article and/or its References.
